# Prostaglandin E_2_ Inhibits Group 2 Innate Lymphoid Cell Activation and Allergic Airway Inflammation Through E-Prostanoid 4-Cyclic Adenosine Monophosphate Signaling

**DOI:** 10.3389/fimmu.2018.00501

**Published:** 2018-03-12

**Authors:** Yu Zhou, Wei Wang, Conghui Zhao, Yan Wang, Haoming Wu, Xiuyuan Sun, Youfei Guan, Yu Zhang

**Affiliations:** ^1^Department of Immunology, School of Basic Medical Sciences, Peking University Health Science Center, Key Laboratory of Medical Immunology, Ministry of Health (Peking University), Beijing, China; ^2^Department of Oral Pathology, Beijing Stomatological Hospital, Capital Medical University, Beijing, China; ^3^Advanced Institute for Medical Sciences, Dalian Medical University, Dalian, China; ^4^Institute of Biological Sciences, Jinzhou Medical University, Jinzhou, China

**Keywords:** group 2 innate lymphoid cell, prostaglandin E_2_, E-prostanoid 4, asthma, IL-33, cyclic adenosine monophosphate

## Abstract

Evidence is accumulating that group 2 innate lymphoid cells (ILC2) play an important role in allergic airway inflammation by producing a large amount of type 2 cytokines. But it remains poorly understood how its activities are properly controlled *in vivo*. Here, we demonstrated that prostaglandin E_2_ (PGE_2_) had a profound inhibitory effect on IL-33-induced ILC2 expansion and IL-5 and IL-13 production *in vitro*. This effect was mimicked by PGE_1_-alcohol but attenuated by ONO-AE3-208, indicating a selective action through the E-prostanoid 4 (EP4) receptor. In the IL-33-induced asthma model, coadministration of PGE_2_ or PGE_1_-alcohol resulted in diminished IL-5 and IL-13 production, reduced eosinophilia and alleviated lung pathology. In contrast, EP4-deficient mice displayed an exacerbated inflammatory response in another ILC2-mediated asthma model induced by *Alternaria* extract. Mechanistic studies demonstrated that the PGE_2_-mediated inhibition of ILC2 was dependent on cyclic adenosine monophosphate (cAMP) production. Further downstream, PGE_2_-EP4-cAMP signaling led to suppression of GATA3 and ST2 expression, which is known to be critical for ILC2 activation. These findings reveal a novel function of PGE_2_ as a negative regulator of ILC2 activation and highlight an endogenous counter-regulatory mechanism for the control of innate allergic inflammatory responses.

## Introduction

Asthma is a chronic inflammatory disease of the airways, which affects over 300 million people worldwide. The inflammatory process in asthma is characterized by eosinophil infiltration, airway hyperresponsiveness, and a chronic T helper 2 (Th2) cell-mediated immune response (1). The type 2 cytokines such as IL-4, IL-5, and IL-13 are key players in the pathogenesis of asthma. More specifically, IL-4 promotes Th2 polarization and IgE class switching in B cells (2, 3). IL-5 drives eosinophilia in lung tissues by enhancing the development of eosinophils in the bone marrow and their recruitment to the lung (1). And IL-13 is found to be necessary and sufficient for mounting airway hyperreactivity and for mucus overproduction (4).

T helper 2 cells have been long considered as the major source of type 2 cytokines in asthma and little is known about the role of innate immunity in the pathology of asthma until the recent discovery of innate lymphoid cells (ILC) (5–7). ILCs share a lymphoid morphology but lack specific antigen receptors and lineage markers. Three groups of ILC have been defined on the basis of their cytokine secretion profiles. Among them, the group 2 ILC (ILC2) is featured by the capacity to produce large amounts of IL-5 and IL-13 in response to epithelial cell-derived cytokines such as IL-33, IL-25, and thymic stromal lymphopoietin (TSLP) (8, 9). In addition to a role in protective immunity against parasites, evidence is accumulating that ILC2 participates in the pathogenesis of allergic inflammation. Increased numbers of ILC2 have been observed in inflamed tissues, such as allergic lung tissues in mice (10), and nasal polyps and skin in human subjects (11–13). In fact, ILC2 represents more than half of the IL-5- and IL-13-producing cells in the lungs of mice subjected to ovalbumin (OVA)- or house dust mite (HDM)-induced asthma. The implication of ILC2 in asthma is further demonstrated in various mouse models of asthma induced by *Alternaria* species, papain, HDM, OVA, and α-GalCer. In these models, ILC2 provokes hyperresponsiveness and goblet cell hyperplasia accompanied with increased eosinophil counts and type 2 cytokines independent of acquired immunity (10, 14–18).

Apart from IL-33, IL-25, and TSLP, ILC2 function is regulated by other cytokines or lipid mediators. TNF superfamily member TL1A, transforming growth factor β (TGF-β), and IL-1 have been shown to enhance the proliferation, type 2 cytokine secretion and/or chemoactivity of ILC2 (19–21). Mice deficient for DR3 (TL1A receptor) or lacking epithelial-derived TGF-β1 exhibit diminished airway inflammation after challenge with allergens. In contrast, both type I and type II interferon (IFN) and IL-27 are able to suppress ILC2 function in a manner dependent on the transcription factor STAT1. Accordingly, ILC2-mediated lung inflammation is enhanced in the absence of the IFN-γ receptor *in vivo* (22–24). More intriguingly, ILC2 appears to be functionally plastic, adapting to environmental cues. IL-12, for example, is able to convert IL-1-primed ILC2 into IFN-γ-producing ILC1 cells, and this transdifferentiation is reversed by IL-4 (25). As for lipid mediators, ILC2 expresses high levels of CRTH2, a receptor for prostaglandin (PG) D_2_, and CysLTR1, the high-affinity receptor for leukotriene D4 (LTD_4_). Both PGD_2_ and LTD_4_ provide activating signals for ILC2, which are nonredundant with IL-33. Defective ILC2-mediated type 2 immune responses are observed in the absence of either PGD_2_ or LT signaling (26–29). Unlike PGD_2_, PGI_2_ serves as a negative regulator for ILC2 function. IL-5 and IL-13 production by IL-33-stimulated ILC2 is potently inhibited by PGI_2_, whereas deficiency in PGI_2_ receptor IP leads to exacerbated lung inflammatory response to the *Alternaria* extract (30). Lipoxin A_4_ (LXA_4_), another arachidonic acid metabolite, also appears to exert a suppressive effect on cytokine production by ILC2 through the unconventional ALX receptor. Notably, severe asthma is associated with decreased expression of LXA_4_ and its receptor ALX (31).

Prostaglandin E_2_ is one of the most abundantly produced PGs. By acting on one or more receptor subtypes [E-prostanoid (EP) 1–4], PGE_2_ affects multiple aspects of immune and inflammatory responses (32). Nevertheless, little is known about its impact on ILC cells except for a recent report in which PGE_2_ has been shown to promote the proliferation and IL-22 production of ILC3 (33). The present study was focused on the role of PGE_2_ in ILC2 biology. Both IL-33-induced activation of ILC2 and ILC2-mediated lung inflammation were found to be profoundly inhibited by PGE_2_-EP4 signaling, most likely through downregulation of GATA3 and ST2.

## Results

### PGE_2_ Inhibits IL-33-Induced Expansion and Type 2 Cytokine Production of Pulmonary ILC2 Cells

**Figure 1:**
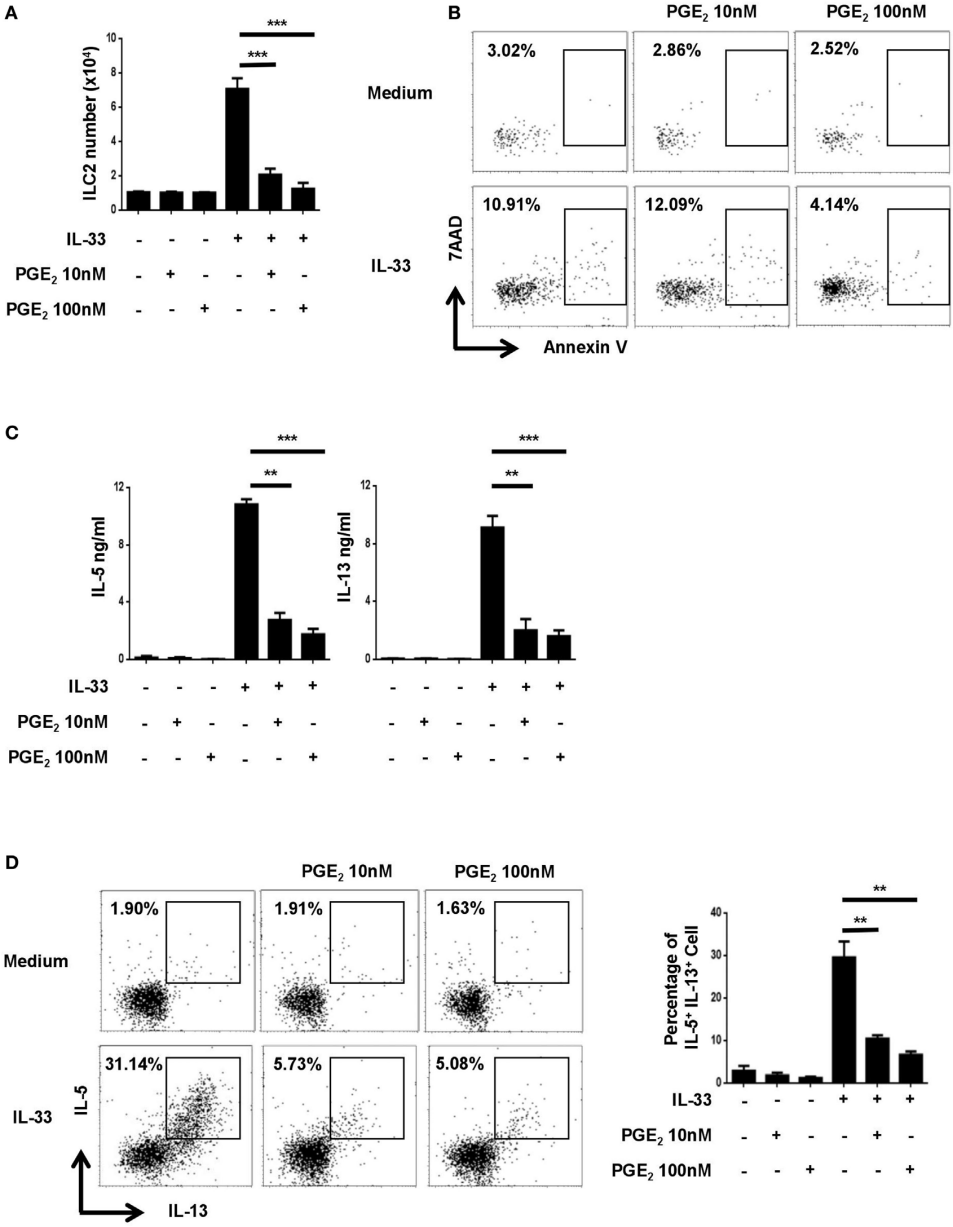
Inhibition of IL-33-induced innate lymphoid cell (ILC) 2 expansion and cytokine production by prostaglandin E_2_ (PGE_2_). Purified ILC2 cells were cultured with or without IL-33 (20 ng/ml) in the presence or absence of 10 or 100 nM PGE_2_. The cultures were analyzed on day 6. **(A)** The number of live cells harvested from the cultures. **(B)** Annexin V and 7AAD staining of total cells from the cultures. **(C)** IL-5 and IL-13 levels in the supernatant as measured by ELISA. **(D)** Intracellular staining for IL-5 and IL-13 production by ILC2. Representative dot plots (*left*) and the percentage of IL-5^+^IL-13^+^ cells (*right*) are shown. Each experiment was repeated at least three times with duplicates for each group. Data are presented as mean ± SD unless otherwise indicated. **p < 0.01 and ***p < 0.001.

To explore the potential impact of PGE_2_ on the activities of ILC2, murine lung ILC2 was isolated by FACS and cultured under various conditions. As previously reported (34), ILC2 cells stimulated with IL-33 underwent drastic expansion and produced a large amount of IL-5 and IL-13 (Figures 1A,C). While PGE_2_ displayed no apparent effect on resting ILC2, it markedly inhibited IL-33-induced ILC2 activation. Specifically, IL-33-induced cell expansion was almost completely abolished with the addition of 10–100 nM PGE_2_ (Figure 1A). As Annexin V/7AAD staining revealed comparable levels of apoptotic cells in IL-33-stimulated cultures with or without PGE_2_ (Figure 1B), the reduced cell number in the presence of PGE_2_ most likely resulted from impaired cell proliferation rather than altered cell viability. When cytokine production was assessed, a similar inhibitory effect was observed with an 70% reduction in both IL-13 and IL-5 in the supernatant of PGE_2_-treated cultures (Figure 1C). This decrease was not simply due to fewer ILC2 cells in the cultures. Intracellular staining showed that PGE_2_ caused a dose-dependent reduction of IL-5- and IL-13-producing cells in total ILC2 cells (Figure 1D), suggesting that they were functionally inhibited as well. Collectively, these *in vitro* studies demonstrate that PGE_2_ acts as a negative regulator for ILC2 activation.

### EP4 Mediates the Inhibitory Effect of PGE_2_ on ILC2

**Figure 2:**
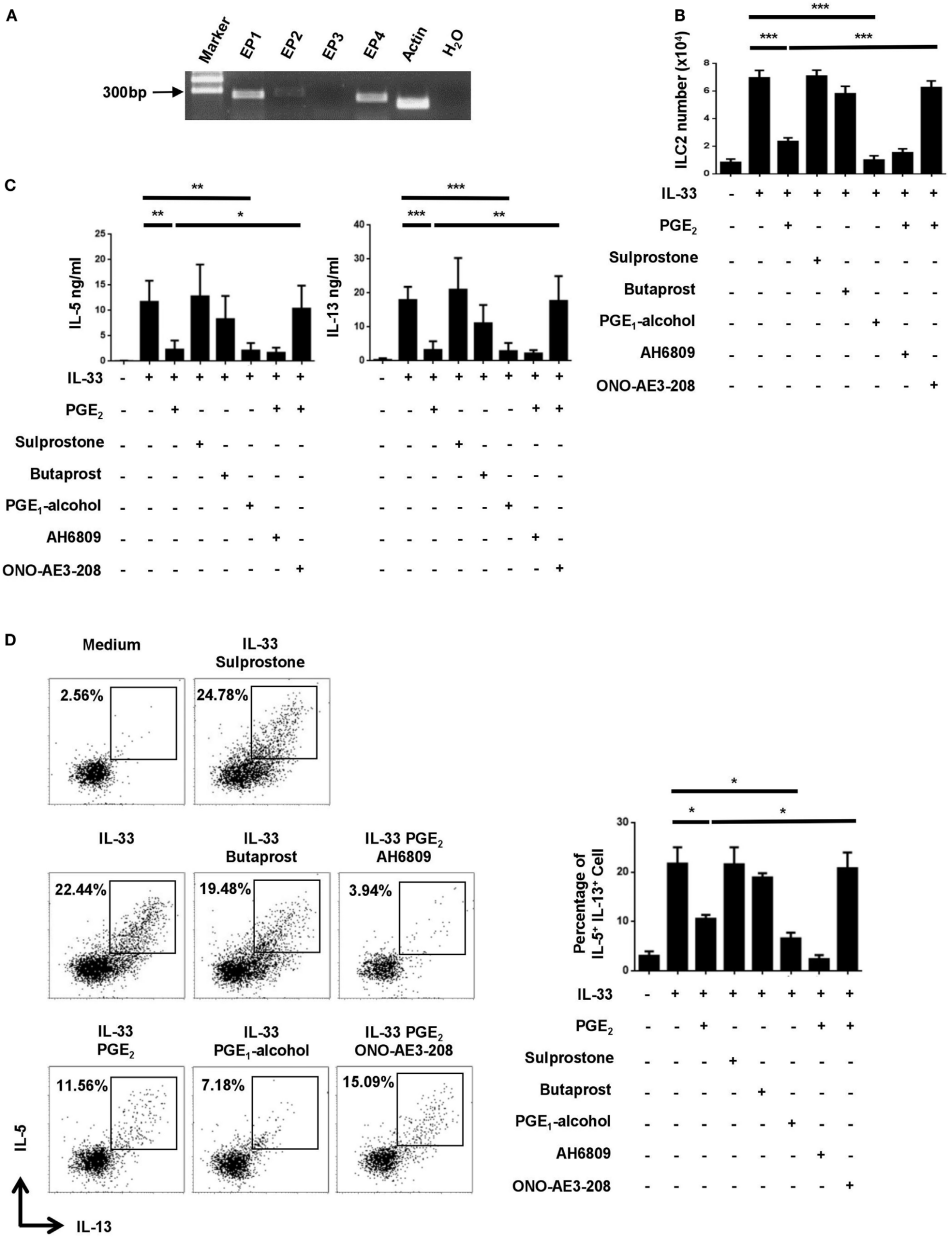
Implication of the E-prostanoid 4 (EP4) receptor in prostaglandin (PG) E_2_-mediated inhibition of innate lymphoid cell 2 (ILC2). **(A)** mRNA expression for each of the four PGE_2_ receptor subtypes in purified ILC2 cells as determined by RT-PCR. Representative results from one out of three independent experiments are shown. **(B–D)** Purified ILC2 cells were stimulated with IL-33 (20 ng/ml) in the presence or absence of PGE_2_ (10 nM), sulprostone (1 μM), butaprost (1 μM), PGE_1_-alcohol (1 μM), AH6809 (10 μM), and/or ONO-AE3-208 (10 μM). The cultures were analyzed on day 6. Live cells were numerated by trypan blue exclusion **(B)**. IL-5 and IL-13 levels in the supernatant were measured by ELISA **(C)**. IL-5 and IL-13-secreting cells were detected by intracellular staining **(D)**. Representative dot plots (*left*) and the percentage of IL-5^+^IL-13^+^ cells (*right*) are shown. Each experiment was repeated at least three times with duplicates for each group. Data are presented as mean ± SD unless indicated otherwise. *p < 0.05; **p < 0.01; and ***p < 0.001.

Prostaglandin E_2_ signals through one of the four functionally distinct receptors, designated EP1, EP2, EP3, and EP4 (32). Their expression in ILC2 was examined by RT-PCR. While *Ptger3* (EP3) mRNA was hardly detected in ILC2, various levels of expression were seen for the other three receptors, with relatively high abundance for *Ptger1* (EP1) and *Ptger4* (EP4) (Figure 2A). To determine which receptor was responsible for PGE_2_-induced inhibition of ILC2, we tested the impact of multiple receptor-selective agonists and antagonists on IL-33-stimualted cultures. EP4 agonist PGE_1_-alcohol was capable of fully mimicking PGE_2_ in the assays. As such, similar inhibition was achieved with PGE_2_ and PGE_1_-alcohol for IL-33-driven ILC2 expansion as shown by cell counts (Figure 2B) and for IL-5 and IL-13 production as determined by either ELISA (Figure 2C) or intracellular staining (Figure 2D). Sulprostone (EP1/EP3 agonist) and butaprost (EP2 agonist), on the other hand, showed no effect (Figures 2B–D). In further support of the key role of EP4, the inhibitory effect of PGE_2_ was abrogated with EP4 antagonist ONO-AE3-208 but not with EP1/2/3 antagonist AH6809. Of note, neither the agonists nor the antagonists had significant impact on cell viability (Figure S1 in Supplementary Material). These results suggest that PGE_2_ primarily signals through the EP4 receptor in ILC2.

### PGE_2_ Suppresses IL-33-Induced Type 2 Responses *In Vivo*

**Figure 3:**
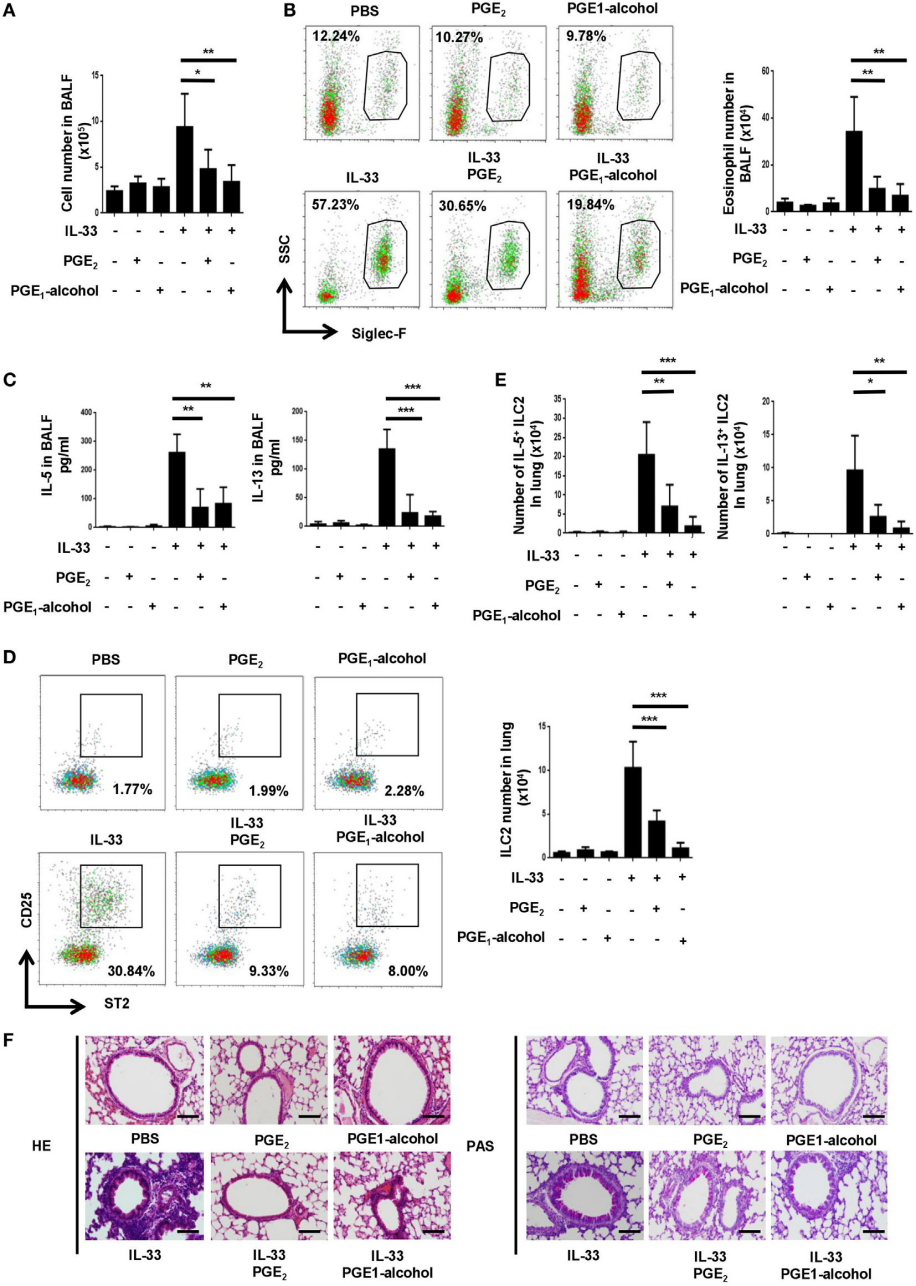
Attenuation of IL-33-induced airway inflammation by prostaglandin (PG) E_2_ and E-prostanoid 4 (EP4) agonist. Mice were treated with IL-33 on days 0 and 2 with or without concomitant administration of PGE_2_ or PGE_1_-alcohol. Airway inflammatory responses were analyzed 24 h after the last challenge. **(A)** Total cell number in the bronchoalveolar lavage fluid (BALF). **(B)** Eosinophils in the BALF were detected by Siglec-F staining. Representative dot plots (*left*) and the number of Siglec-F^+^ cells (*right*) are shown. **(C)** Levels of IL-5 and IL-13 in BALF were measured by ELISA. **(D)** Innate lymphoid cell 2 (ILC2) cells in the lung tissue were detected by CD25 and ST2 staining in the Lin^-^CD45^+^ gate. Representative dot plots (*left*) and the number of CD25^+^ST2^+^ cells (*right*) are shown. **(E)** The percentage of IL-5^+^ or IL-13^+^ cells among pulmonary ILC2 cells as determined by intracellular staining. **(F)** Representative images showing hemotoxylin and eosin (HE) and Periodic Acid-Schiff (PAS) staining of the lung tissue. Scale bar equals 100 µm. The experiments were repeated at least three times with four mice for the control, PGE_2_ and PGE_1_-alcohol group and six mice for the IL-33, IL-33 + PGE_2_ and IL-33 + PGE_1_-alcohol group. Data are presented as mean ± SD unless indicated otherwise. *p < 0.05; **p < 0.01; and ***p < 0.001.

In view of its potent inhibitory function *in vitro*, we sought to determine whether PGE_2_ affected ILC2-mediated airway inflammation induced by IL-33 (22, 23). Intranasal administration of IL-33 induced rapid and robust type 2 responses characterized by ILC2 accumulation, type 2 cytokine secretion, eosinophilia and goblet cell metaplasia. Such responses were largely diminished when PGE_2_ was coadministrated with IL-33. In comparison to mice treated with IL-33 alone, those receiving IL-33 + PGE_2_ showed approximately 50% reduction in total cells (Figure 3A) and 70% reduction in Siglec-F^+^ eosinophils (Figure 3B) in the bronchoalveolar lavage fluid (BALF). In addition, coadministration of PGE_2_ led to approximately 60% reduction of both IL-5 and IL-13 in the lavage (Figure 3C). As much as the ILC2 population was concerned, a much diminished accumulation was detected in the lung tissue of PGE_2_-treated mice (Figure 3D). Moreover, ILC2 recovered from these mice contained a significantly lower percentage of IL-5- or IL-13-producing cells (Figure 3E; Figure S2A in Supplementary Material). Furthermore, hemotoxylin and eosin (HE) and Periodic Acid-Schiff (PAS) staining of the lung tissue demonstrated that coadministration of PGE_2_ ameliorated IL-33-induced lung pathology featured by intensive peribronchial lymphoid infiltration and goblet cell metaplasia (Figure 3F).

CD4^+^ T cells represent another important source of type 2 cytokines. To formally exclude their contribution to the airway inflammation induced by short-term treatment with IL-33, flow cytometry was performed to analyze cytokine production by CD4^+^ T cells. In agreement with previous studies (30), IL-5- or IL-13-producing cells were barely detectable among CD4^+^ cells in the lung tissue following IL-33 administration, irrespective of the presence or absence of PGE_2_ (Figure S2B in Supplementary Material). Therefore, PGE_2_ primarily exerts its anti-inflammatory effect by acting on ILC2 cells in such a scenario.

To verify the role of EP4 in PGE_2_-mediated inhibition *in vivo*, a group of mice received EP4 agonist PGE_1_-alcohol in conjunction with IL-33. PGE_1_-alcohol displayed equal potency to PGE_2_ in the inhibition of IL-33-indcued airway inflammation in all aspects examined, including exudation of eosinophils and other inflammatory cells, accumulation of ILC2, production of IL-5 and IL-13, and lung pathology (Figures 3A–E).

### EP4-Deficiency Leads to Exaggerated Asthmatic Responses to *Alternaria* Extract

**Figure 4:**
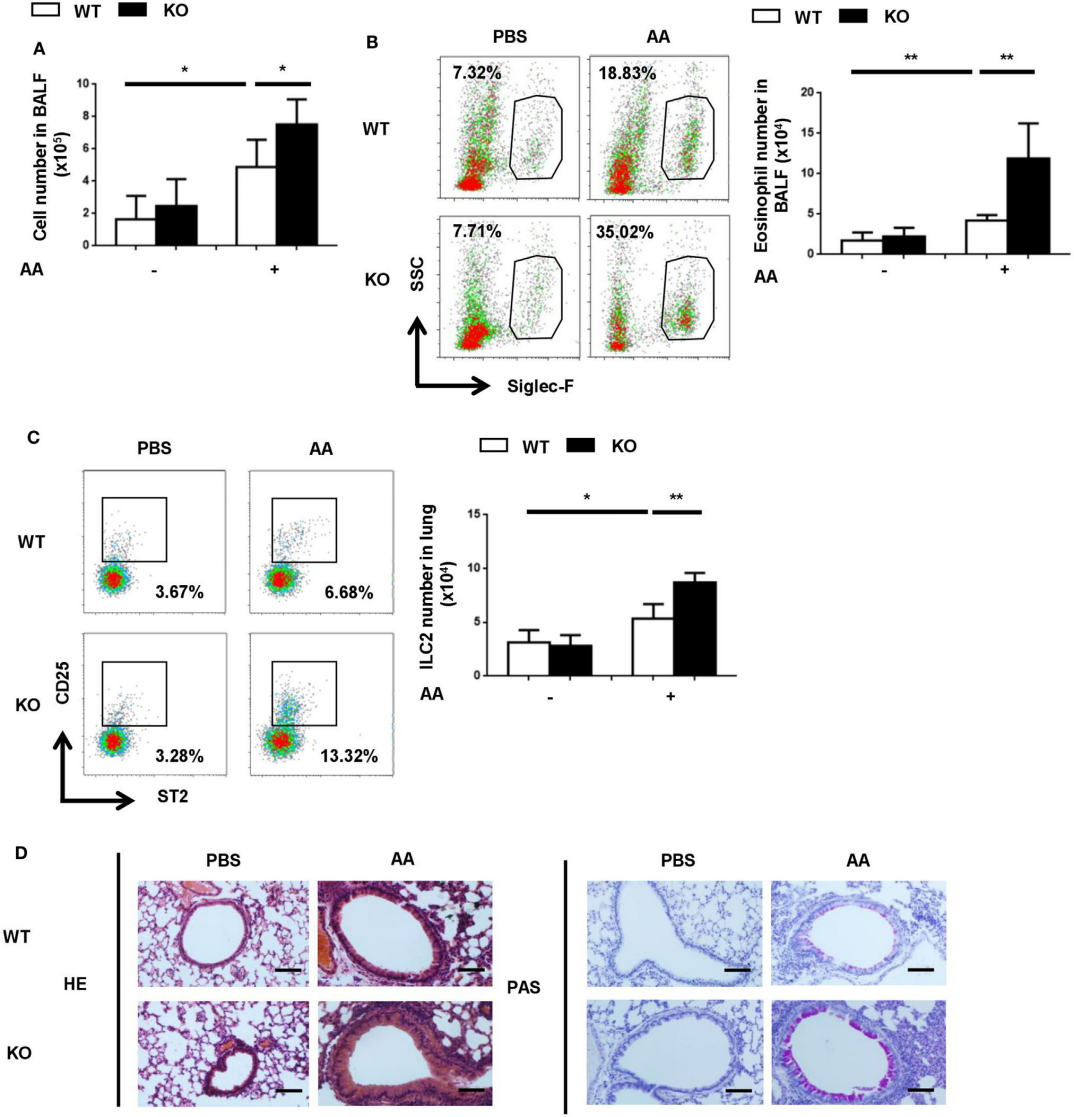
Exacerbated airway inflammatory responses to *Alternaria* extract in E-prostanoid 4 (EP4)-deficient mice. Wild-type (WT, white column) and *Ptger4*^flox/flox^-Vav-Cre (KO, black column) mice were challenged with *Alternaria Alternata* (AA) on days 0, 3, and 6. Airway inflammatory responses were analyzed on day 8. **(A)** Total cell number in the BALF. **(B)** Representative Siglec-F staining (*Left*) and the number of Siglec-F^+^ eosinophils (*Right*) in the BALF. **(C)** Innate lymphoid cell 2 (ILC2) cells in the lung tissue were detected by CD25 and ST2 staining in the Lin^-^CD45^+^ population. Representative dot plots (*left*) and the number of CD25^+^ST2^+^ cells (*right*) are shown. **(D)** Representative images showing hemotoxylin and eosin (HE) and Periodic Acid-Schiff (PAS) staining of the lung tissue. Scale bar equals 100 µm. The experiments were repeated at least three times with five mice for each group. Data are presented as mean ± SD unless indicated otherwise. *p < 0.05; **p < 0.01; and ***p < 0.001.

Next, we explored whether endogenous PGE_2_ signal participated in the control of ILC2-mediated airway inflammation. Given the key role of EP4 in PGE_2_ signaling in ILC2 cells, it was anticipated that its deficiency might resulted in aggravated inflammation. To test this, *Ptger4*^flox/flox^mice were crossed with Vav-Cre mice to specifically delete *Ptger4* in hematopoietic cells. The deletion efficiency was verified by the greatly reduced expression of *Ptger4* mRNA in total bone marrow cells and CD4^+^ T cells in knockout versus wild-type mice (Figure S3 in Supplementary Material). We first compared the wild-type and knockout mice in the IL-33-induced asthma model and found no overt difference between them. In consideration of the possibility that the overwhelming response to IL-33 might prevent the manifestation of potential aggravating effects of EP4 deficiency, we switched to a clinically more relevant model induced by the *Alternaria* extract, for which ILC2 is known to be a key player (35, 36). In comparison to the rapid and potent inflammatory response induced by IL-33, a milder type 2 response was observed following the airway challenge with the *Alternaria* extract. The numbers of total inflammatory cells in the BALF and ILC2 cells in the lung tissue were reduced by half (Figures 4A,C in comparison with Figures 3A,D). An even dramatic difference was seen in terms of eosinophils in the BALF (Figure 4B in comparison with Figure 3B). This allowed us to assess any inflammation-promoting effect introduced by EP4 deficiency. Indeed, the EP4-deficient mice showed increased numbers of ILC2, eosinophils and other inflammatory cells when compared to wild-type mice after challenge with the *Alternaria* extract (Figures 4A–C). Moreover, histological analyses demonstrated more intensive peribranchial lymphoid infiltration and increased mucus production (Figure 4D). These results indicate that the endogenous PGE_2_-EP4 signal is actively involved in the regulation of ILC2-mediated airway inflammation.

### PGE_2_-EP4-Cyclic Adenosine Monophosphate (cAMP) Signaling Inhibits the Expression of GATA3 and ST2

**Figure 5:**
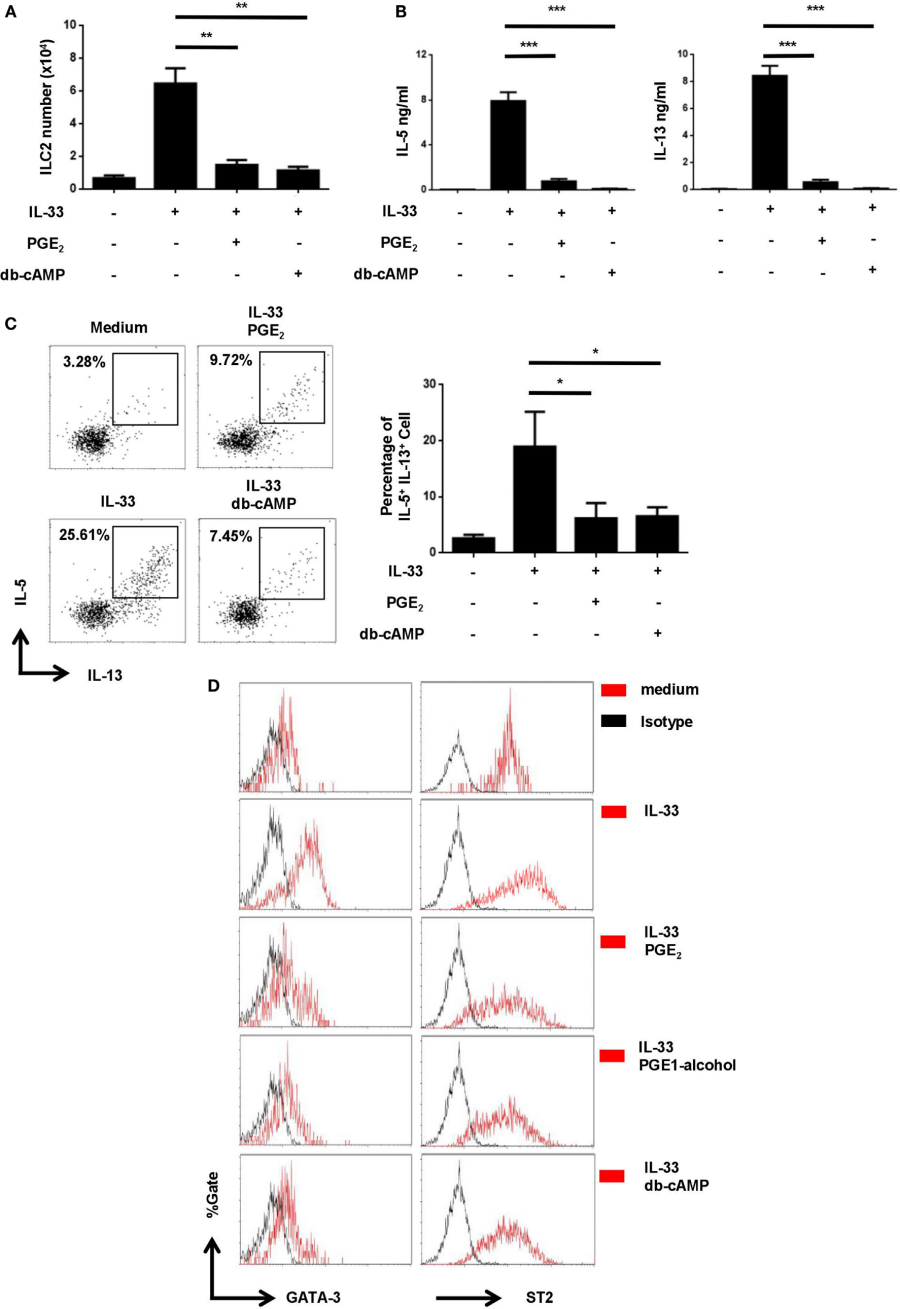
Inhibition of IL-33-induced upregulation of GATA3 and ST2 by prostaglandin E_2_ (PGE_2_)- E-prostanoid 4 (EP4)- cyclic adenosine monophosphate (cAMP) signaling. Purified innate lymphoid cell (ILC) 2 cells were stimulated with IL-33 (20 ng/ml) in the presence or absence of PGE_2_ (10 nM), PGE_1_-alcohol (1 μM), or dibutyryl cAMP (db-cAMP) (400 µM) and the cultures were analyzed on day 6. **(A)** The number of live cells harvested from the cultures. **(B)** IL-5 and IL-13 levels in the supernatant as measured by ELISA. **(C)** Intracellular staining for IL-5 and IL-13 production by ILC2. Representative histograms (*left*) and the percentage of IL-5^+^IL-13^+^ cells (*right*) are shown. **(D)** Intracellular staining for GATA3 (*left*) and ST2 (*right*). Representative histograms are presented with isotype controls (dash line). Each experiment was repeated at least three times with duplicates for each group. Data are presented as mean ± SD unless indicated otherwise. *p < 0.05; **p < 0.01; and ***p < 0.001.

E-prostanoid 4 has been shown to signal through two main pathways, either by triggering cAMP production and subsequent activation of PKA (37) or by engaging the PI3K-Akt pathway (38). To determine which one was involved in ILC2 inhibition, we tested small molecular compounds specifically targeting the two pathways. Dibutyryl cAMP (db-cAMP), a cell-permeable cAMP analog, was able to simulate PGE_2_ in ILC2 cultures, leading to drastic inhibition of the IL-33-driven cell expansion (Figure 5A) and IL-5/IL-13 production (Figures 5B,C). On the other hand, the suppressive activity of PGE_2_ remained unaltered in the presence of the PI3K inhibitor LY294002 (39) (Figure S4 in Supplementary Material). Therefore, PGE_2_ primarily functions through the cAMP pathway instead of the PI3K pathway in ILC2 cells.

Similar to Th2 cells, ILC2 relies on the transcription factor GATA3 for its differentiation as well as the maintenance of its functionality (40). In Th2 cells, IL-33 has been shown to enhance GATA3 expression, which in turn leads to elevated levels of ST2, a component of IL-33 receptor (41). We wondered whether IL-33 had a similar effect in ILC2 and how it was modulated by the PGE_2_-EP4-cAMP signaling. Purified ILC2 cells were thus stimulated with IL-33 in the presence or absence of PGE_2_, PGE_1_-alcohol, or db-cAMP and examined for GATA3 and ST2 expression by intracellular staining. As shown in Figure 5D, exposure to IL-33 resulted in an elevated level of GATA3 in ILC2. Further reminiscent of its effect in Th2 cells, IL-33 also induced the upregulation of ST2 expression by ILC2 (Figure 5D). Intriguingly, the augmented expression of both GATA3 and ST2 was found to be abrogated with the addition of PGE_2_, PGE_1_-alcohol, or db-cAMP (Figure 5D). Therefore, there appears to be a self-amplification mechanism in IL-33 signaling in ILC2 and PGE_2_-EP4-cAMP signaling imposes a break for the positive feedback loop.

## Discussion

The present study revealed a novel function of PGE_2_ as a negative regulator of ILC2 activation. When added to ILC2 cultures, PGE_2_ profoundly inhibited IL-33-induced cell expansion and type 2 cytokine production in a dose-dependent manner. Moreover, intranasal administration of PGE_2_ prevented ILC2 accumulation in the lung and attenuated airway inflammation in an asthma model induced IL-33. As the inhibitory effect was fully mimicked by PGE_1_-alcohol but reverted by a EP4-selective antagonist, PGE_2_ appeared to signal exclusively through EP4 in ILC2. In agreement with this speculation, EP4-deficient mice displayed an exacerbated inflammatory response to the airway challenge of *Alternaria* extract. We went further to demonstrate that the PGE_2_-EP4 signaling was primarily mediated by cAMP, which in turn suppressed IL-33-induced upregulation of GATA3, a master transcriptional regulator for the ILC2 lineage.

An increasing body of evidence indicates an important role of lipid mediators, especially those derived from arachidonic acid in orchestrating ILC2 functions under physiological and pathological conditions (42). Arachidonic acid is generated from cell membrane phospholipids by phospholipase A_2_ and subsequently converted into leukotrienes by 5-lipoxygenase, lipoxins by 15-lipoxygenase, or PGE_2_, PGI_2_, PGD_2_, PGF_2α_, and thromboxane A2 by cyclooxygenase (COX)-1/2 and specific PG synthase through two unstable intermediates PGG_2_ and PGH_2_ (43). PGD_2_ and LTD_4_ are potent activators of ILC2, triggering production of type 2 cytokines on their own or through synergism with IL-25/33 by inducing intracellular Ca^2+^ mobilization (26–29). PGI_2_ and LXA_4_, on the other hand, attenuate ILC2 responses to IL-33 *via* inducing cAMP production and ERK phosphorylation, respectively (30, 31). Here, we reported the identification of PGE_2_, another metabolite of arachidonic acid, as a novel endogenous inhibitor for the activation of murine ILC2 cells. Of note, while this manuscript was in preparation, Maric et al. published their studies of PGE_2_ on human ILC2, in which PGE_2_ was shown to exert a similar inhibitory effect (44). Their studies were based exclusively on *in vitro* and pharmacological approaches. The present study, on the other hand, also provided *in vivo* and genetic evidence for PGE_2_-mediated inhibition of ILC2 and its implication in airway inflammation. Interestingly, While PGE_2_ primarily signals through the EP4 receptor in murine ILC2, both EP2 and EP4 are involved in the response of human ILC2 to PGE_2_. The species difference may partly result from the much lower expression of EP2 in murine ILC2.

Prostaglandin E_2_ is known for its diverse and sometimes paradoxical activities on different immune cells at different stages of immune responses (32). For example, while imposing an overall suppressive effect on T cell activation and production, it differentially regulates the lineage specification of CD4^+^ effector T cells, shifting the balance away from type 1 toward other forms of immunity, such as Th2, Th17, and Treg responses (39, 45–47). With respect to ILC cells, a recent study by Duffin et al. has demonstrated that PGE_2_-EP4 signaling promotes the homeostasis and IL-22 production of ILC3. Our finding that PGE_2_ antagonizes IL-33-induced ILC2 expansion and cytokine production indicates additional complexity of PGE_2_-mediated immunoregulation.

Prostaglandin E_2_ and PGI_2_ display similar yet distinct properties in their action on ILC2. While they both profoundly inhibit ILC2 proliferation and cytokine production, increased cell death is only observed in PGI_2_ but no in PGE_2_ cultures. Moreover, although a cAMP-dependent mechanism is shared by PGE_2_ and PGI_2_, further downstream targets appear to differ from each other. PGI_2_ causes a dramatic reduction of Id2 mRNA expression (30), whereas PGE_2_-mediated ILC2 inhibition is associated with a marked decrease in GATA3 at the protein level. This discrepancy may be partly explained by the different receptors engaged by PGI_2_ and PGE_2_. It is well established that GATA3 is essential for early ILC2 development. Besides, sustained GATA3 activity is required for the maintenance and functionality of ILC2 (40, 48). Little is known, however, about the mechanisms governing GATA3 expression in ILC2. A recent study has reported that activation of resting ILC2 by TSLP increased the expression of GATA3 (49). We found that GATA3 expression was also enhanced in ILC2 after stimulation with IL-33. This is consistent with two previous reports in which IL-33 has been shown to upregulate GATA3 expression in Th2 and Treg cells, respectively (41, 50). In the latter case, treatment with IL-33 even induces the conversion of Treg cells into Th2-like cells (50). Of note, IL-33 stimulation also leads to enhanced expression of its receptor component ST2 in Th2, Treg as well as ILC2 cells. The study by Guo et al. further reveals that GATA3 is directly involved in ST2 upregulation in IL-33 stimulated Th2 cells (41). Taken together, there appears to be a self-amplification mechanism in IL-33 signaling; IL-33 induces GATA3 expression, which in turn results in elevated levels of ST2 and increased responsiveness to IL-33. Despite its apparent beneficial effects, such a mechanism can be problematic if left unchecked. Data shown here indicate that the PGE_2_-EP4-cAMP signaling may serve as a break to prevent hyperactivation of ILC2. Antagonism against IL-33-induced GATA3 expression is centrally positioned in its action. A decrease in GATA3 activity not only directly affects the expression of effector cytokines including IL-5 and IL-13, but also disrupts the self-amplification loop of IL-33 signaling as a result of ST2 suppression. Nevertheless, the actual contribution of reduced GATA3 expression to inhibition of ILC activation remains to be experimentally verified, for example, by observing whether enforced GATA3 expression renders ILC2 resistant to PGE_2_-mediated inhibition, although it is technically challenging due to the limited number of ILC2 cells available. Further studies are also warranted to unravel the mechanisms underlying cAMP-induced reduction of GATA3 expression in ILC2. One candidate could be NF-κB activation, which is known to be important for GATA3 expression and Th2 cell differentiation (51). In view of the striking similarities between the transcriptional programs controlling Th2 and ILC2 cells, it would not be surprising if NF-κB plays a similar role in ILC2.

Prostagladins, especially PGD_2_ and PGE_2_, have been intensively studied for their implications in asthmatic inflammation. While it is generally agreed that PGD_2_ accelerates the development of asthma (52, 53), data are controversial regarding the role of PGE_2_ in the pathogenesis of asthma. In contrast to the heightened inflammatory responses in COX-1- or COX-2-deficient mice (54), mice lacking mPGES1, an enzyme specific for PGE_2_ synthesis, show reduced inflammation in OVA-induced asthma models (55). Another study, however, has demonstrated that mPGES1 deficiency has no significant impact on allergen sensitization and generation of effector T cells but induces marked remodeling of the pulmonary vasculature during chronic exposure to the HDM-derived allergen Der f, indicative of a beneficial effect of PGE_2_ in the late phase of asthmatic inflammation (56). Conflicting results have also been obtained in analyses of mice with selective deletion of genes encoding each of the four PGE_2_ receptors. Kunikata et al. have reported that, while mice lacking EP1, EP2, or EP4 display an allergic response comparable to that of the wild-type littermates, EP3 deficiency causes much more pronounced airway inflammation. In their model, the EP3 signal primarily influences the elicitation stage by suppressing the release of allergic mediators from mast cells and the production of chemokines by epithelial cells (57). Another study demonstrates that PGE_2_ may also exert a protective effect through EP_2_ by targeting CD4^+^ T cells at the sensitization stage (58). Still another study, however, reveals that the EP_2_ signal actually facilitates asthma development, possibly by enhancing IgE production by B cells (59). The discrepancy may be ascribed to the multifaceted activities of PGE_2_ on different cell types at different stages of asthma development. Alternatively, it may be reflective of the nature of asthma as an extraordinarily heterogeneous disease. Two major forms of asthma have been defined in clinic. The atopic asthma is characterized by Th2 cell-mediated responses and IgE production to various allergens. The non-atopic asthma, on the other hand, is independent on adaptive immunity (1). Instead, it is often associated with hyperactivation of ILC2. Consistent with the potent inhibitory activity of PGE_2_-EP4 signaling on ILC2 *in vitro*, intranasal administration of PGE_2_ or the EP4-selective agonist PGE_1_-alcohol attenuates IL-33-induced allergy *in vivo* as demonstrated by the reduced number of eosinophils and other inflammatory cells in BALF and improved histology of the lung tissue. On the other hand, EP4 deficiency results in a more severe airway inflammation when challenged with *Alternaria* extract. These data suggest a proresolving role of PGE_2_ in ILC2-mediated allergic inflammation. It would be interesting to explore any potential defects in the PGE2-EP4 signaling pathway in severe, non-atopic asthma induced, for example, by environmental pollutants, irritants, chronic airway mycosis, or repetitive viral infection.

In conclusion, the present study identifies new cellular targets and mechanisms for PGE_2_ in the regulation of asthma pathobiology. Its anti-inflammatory actions suggest a potential therapeutic strategy in asthma for which ILC2 plays a predominant role.

## Materials and Methods

### Mice

C57BL/6 mice were brought from Vital River Laboratories and were bred in the animal breeding facility at Peking University Health Science Center under specific pathogen-free conditions. To generate mice with selective EP4 deficiency in hematopoietic cells, *Ptger4*^flox/flox^ mice (60) were crossed with Vav-Cre mice (The Jackson Laboratory, line 8610), both on C57BL/6 background. The experimental procedures on use and care of animals had been approved by the Ethics Committee of Peking University Health Science Center.

### Antibodies and Reagents

Biotin-conjugated anti-CD4 (GK1.5), CD8 (53-6.7), CD5 (53-7.3), TCRβ (H57-597), CD11b (M1/70), CD11c (N418), Gr-1 (RB6-8C5), B220 (RA3-6B2), TER119 (TER-119), FcεRIα (MAR-1), APC-conjugated anti-ST2 (RMST2-2), PE-conjugated anti-CD25 (PC61.5), PerCP-Cyanine5.5-conjugated anti-IL-5 (TRFK5), PE-Cyanine7-conjugated GATA-3 (TWAJ), and PE-Cyanine5.5-conjugated anti-IL-13 (eBio13A) were purchased from eBioscience. PE-conjugated anti-CD25 (7D4) and PerCP-Cyanine5.5-conjugated anti-CD45 (30-F11) were purchased from BD Pharmingen. FITC-conjugated and APC-conjugated streptavidin, recombinant mIL-33 were purchased from BioLegend. Recombinant mIL-2 and mIL-7 were purchased from R&D. APC-conjugated anti-ST2 (DJ8) was purchased from MD Bioproducts. PE-conjugated anti-Siglec-F (ES22-10D8) and Streptavidin MicroBeads were purchased from MiltenyiBiotec. PGE_2_, sulprostone, butaprost, PGE_1_-alcohol, AH6809, and ONO-AE3-208 were purchased from Cayman Chemical. Dibutyryl cAMP (db-cAMP) was purchased from Sigma-Aldrich.

### Flow Cytometry

Pulmonary cells were stained for lineage markers (TCRβ, CD4, CD8, CD5, B220, CD11b, CD11c, Gr-1, TER119, FcεRIα), CD45, CD25 and ST2. ILC2 were defined as CD25 and ST2 double positive cells in the Lin^-^CD45^+^gate. Annexin V and 7AAD staining was performed to detect apoptotic cells following recommendations of the manufacturer. Flow cytometric analysis was performed on Gallios Beckman Coulter using Kaluza software. Lung ILC2s were sorted using FACSAria (BD Bioscience).

### Cell Preparation

Bronchoalveolar cells were prepared by lavaging lungs with 2 × 0.5 ml PBS. To obtain whole pulmonary cells, finely chopped lung tissues were incubated in RPMI 1640 containing 0.5 mg/ml DNase I (Sigma Aldrich), 1 mg/ml Collagenase D (Roche) for 1 h at 37°C with gentle shaking. Cell suspensions were obtained by passing through a 100 µm strainer. Mononuclear cells were isolated with a Percoll gradient (GE Healthcare) by harvesting cells at densities between 35 and 70% after centrifugation at 500 *g* for 20 min at room temperature.

### Airway Inflammation Models and Analytical Procedures

To induce airway inflammation, mice were anesthetized with pelltobarbitalum natricum and received intranasal treatment with IL-33 (1 µg per mouse) on day 0 and day 2. The animals were sacrificed 24 h after the last challenge. The *Alternaria* induced asthma model was generated as described (22, 28). Briefly, mice were intranasaly challenged with *Alternaria* extract (100 µg per mouse, Greer Labs) on days 0, 3, and 6. The animals were sacrificed on day 8. BALF was collected after flushing with 2 × 500 μl PBS and analyzed for the presence of inflammatory cells and cytokine levels. The number of ILC2 cells in the lung was determined by flow cytometry. To detect IL-5/IL-13-secreting cells, lung cells were cultured in medium contain 20 ng/ml PMA and 10 µg/ml ionomycin with GolgiStop (BD Bioscience) for 6 h and then analyzed by flow cytometry after surface staining for lineage markers and intracellular staining of IL-5 and IL-13. For histology, lung tissues were fixed in 4% paraformaldehyde and stained following standard protocols for HE and PAS. In some experiments, PGE_2_ (300 mg/kg) and PGE_1_-alcohol (500 mg/kg) was administered concomitantly with IL-33 or *Alternaria* extract.

### Culture of ILC2s and Measurement of Cytokines

Purified pulmonary ILC2s were seeded into 96-well round bottomed cell culture plates and allowed to rest for at least 24 h in RPMI 1640 supplemented with 10% FBS, 0.05 mM 2-mercaptoethanol, 100 µg/ml penicillin and streptomycin, 10 mM HEPES and 10 ng/ml IL-2 and IL-7. The cells were then replated at a density of 2,000–2,500/well and stimulated with 20 ng/ml IL-33 in the absence of IL-2 and IL-7. When applicable, PGE_2_ was added at 10–100 nM, sulprostone, butaprost, PGE_1_-alcohol at 1 μM, AH6809 and ONO-AE3-208 at 10 μM, and db-cAMP at 400 µM. After 6 days culture, the concentration of IL-5 and IL-13 in the supernatant was determined using ELISA Ready-SET-Go! Kits (eBioscience). Live cells were counted by trypan blue staining and apoptosis was analyzed by Annexin V and 7AAD staining. For analysis of cytokine production by individual cells, the culture was stimulated for another 6 h using 20 ng/ml PMA and 10 µg/ml Ionomycin with GolgiStop (BD Bioscience) prior to intracellular staining for IL-5 and IL-13.

### Gene Expression Analysis by PCR

RNA was prepared from sorted ILC2s using the Rneasy Mini Kit (Qiagen). cDNA was synthesized using the AMV cDNA Reverse Transcription Kit (Promega). PCR was performed with primers as follows: EP1 (5′-ATGGTCTTCTTCGGCCTGTG-3′ and 5′-TAAAACACCAGGTGCCAGGG-3′), EP2 (5′-TCACCTTCGCCATATGCTCC-3′ and 5′-GGCACTGGACTGGGTAGAAC-3′), EP3 (5′-TCTGTTGGTCGCCGCTATTG-3′ and 5′-GGTGTGGTCTCTGATCTGGC-3′), EP4 (5′-TTGCTTCCAGGTTCGCATGG-3′ and 5′-AGTTTCACTGGGGAATGTGACT-3′), and β-actin (5′-AGAGGGAAATCGTGCGTGAC-3′ and 5′-CAATAGTGATGACCTGGCCGT-3′).

### Statistics

All the experiments were repeated at least three times. For statistical analysis, paired and unpaired Student’s *t*-test was performed using GraphPad Prism 6 (GraphPad Software). *p < 0.05; **p < 0.01; and ***p < 0.001.

## Ethics Statement

This study was carried out in accordance with the recommendations of Ethics Committee of Peking University Health Science Center. The protocol was approved by the Ethics Committee of Peking University Health Science Center.

## Author Contributions

YZhou, WW, YG, and YZhang designed the project. YZhou and WW did the experiment. YZhou, WW, and YZhang wrote the manuscript. YG applied the EP4flox/flox mice. CZ contributed to the pathology analysis. YW and HW contributed to establish the asthma model. XS contributed to ILC2s sorting.

## Conflict of Interest Statement

The authors declare that the research was conducted in the absence of any commercial or financial relationships that could be construed as a potential conflict of interest. The reviewer HW and handling Editor declared their shared affiliation.

## Supplementary Material

Figure S1 Unaltered cell viability following exposure to PGE_2_, PGE_2_ agonists, and/or PGE_2_ antagonists. ILC2 cell culture was established as described in Figure 2. Cell viability was examined on day 6 by Annexin V and 7AAD staining. Representative dot plots are shown.

Figure S2 IL-5 and IL-13 production by ILC2 versus CD4^+^ T cells in the IL-33-induced asthma models. Airway inflammation was induced as described in Figure 3. IL-5 and IL-13 production was analyzed by intracellular staining after gating on Lin-CD45^+^ST2^+^ ILC2 **(A)** or TCRβ^+^ **(B)** cells. Representative dot plots are shown.

Figure S3 *Ptger4* mRNA expression in total bone marrow cells and CD4^+^ T cells from wild-type (WT) and *Ptger4*^flox/flox^-Vav-Cre (KO) mice as determined by RT-PCR.

Figure S4 Independence of PGE_2_-mediated inhibition on PI3K activation. ILC2 cells were stimulated with IL-33 (20 ng/ml) in the presence or absence of PGE_2_ (10 nM) with or without the addition of PI3K inhibitor LY294002 (10 µM). IL-5 and IL-13 levels in the supernatant were measured by ELISA.

## Abbreviations

BALF, bronchoalveolar lavage fluid; EP1–4, E-prostanoid 1–4; db-cAMP, dibutyryl cyclic adenosine monophosphate; HDM, house dust mite; ILC, innate lymphoid cell; ILC2, group 2 innate lymphoid cell; IFN, interferon; OVA, ovalbumin; PG, prostaglandin; Th2, T helper 2; TSLP, thymic stromal lymphopoietin.
