# Supplementary figures and images for: Prostaglandin E2 Inhibits Group 2 Innate Lymphoid Cell Activation and Allergic Airway Inflammation Through E-Prostanoid 4-Cyclic Adenosine Monophosphate Signaling
Source: Front Immunol. 2018 Mar 12;9:501. doi: 10.3389/fimmu.2018.00501 (PMC5857904; doi:10.3389/fimmu.2018.00501)

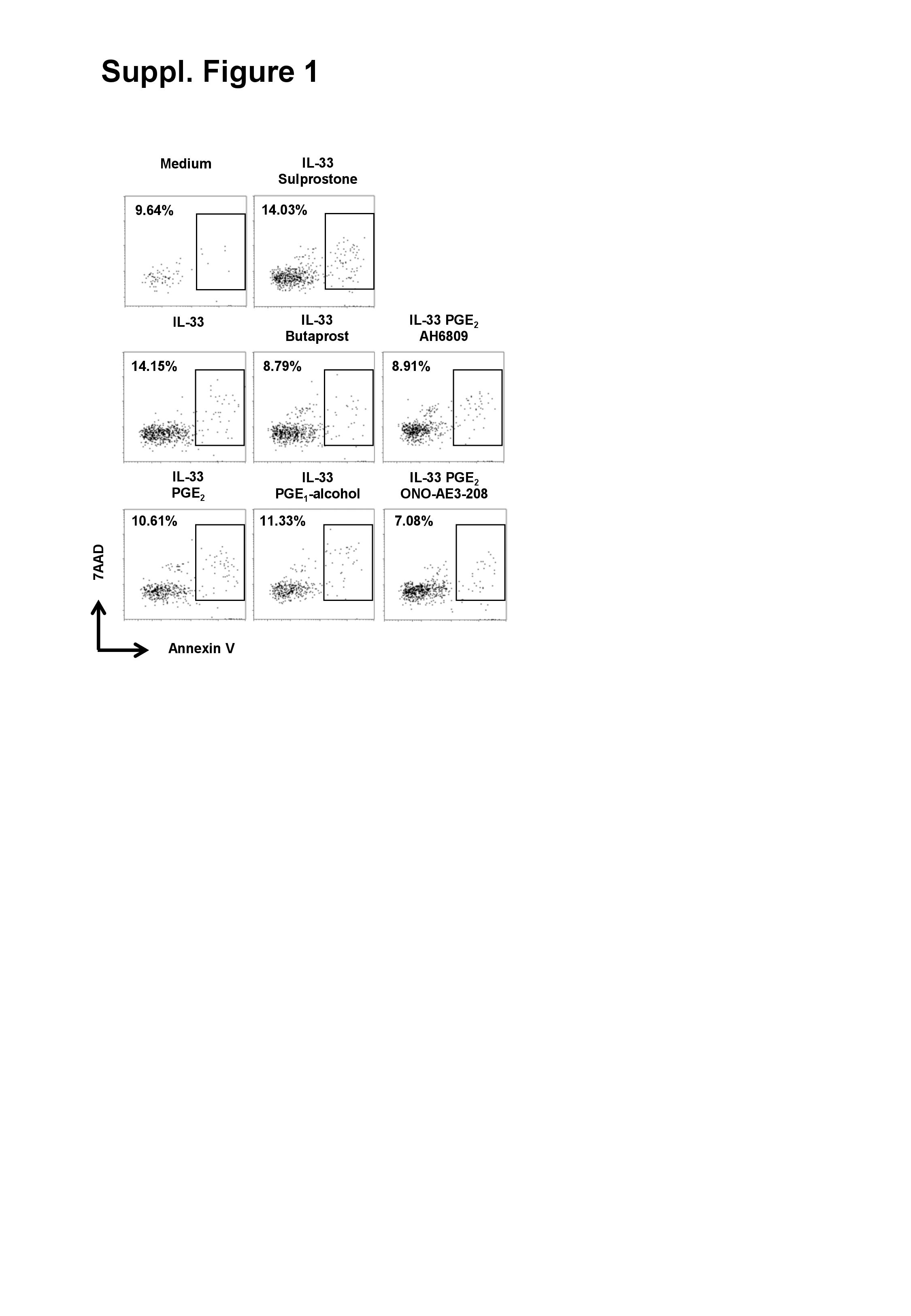

Supplement: Figure S1 — Unaltered cell viability following exposure to PGE2, PGE2 agonists, and/or PGE2 antagonists. ILC2 cell culture was established as described in Figure 2. Cell viability was examined on day 6 by Annexin V and 7AAD staining. Representative dot plots are shown. [file image_1.jpg]

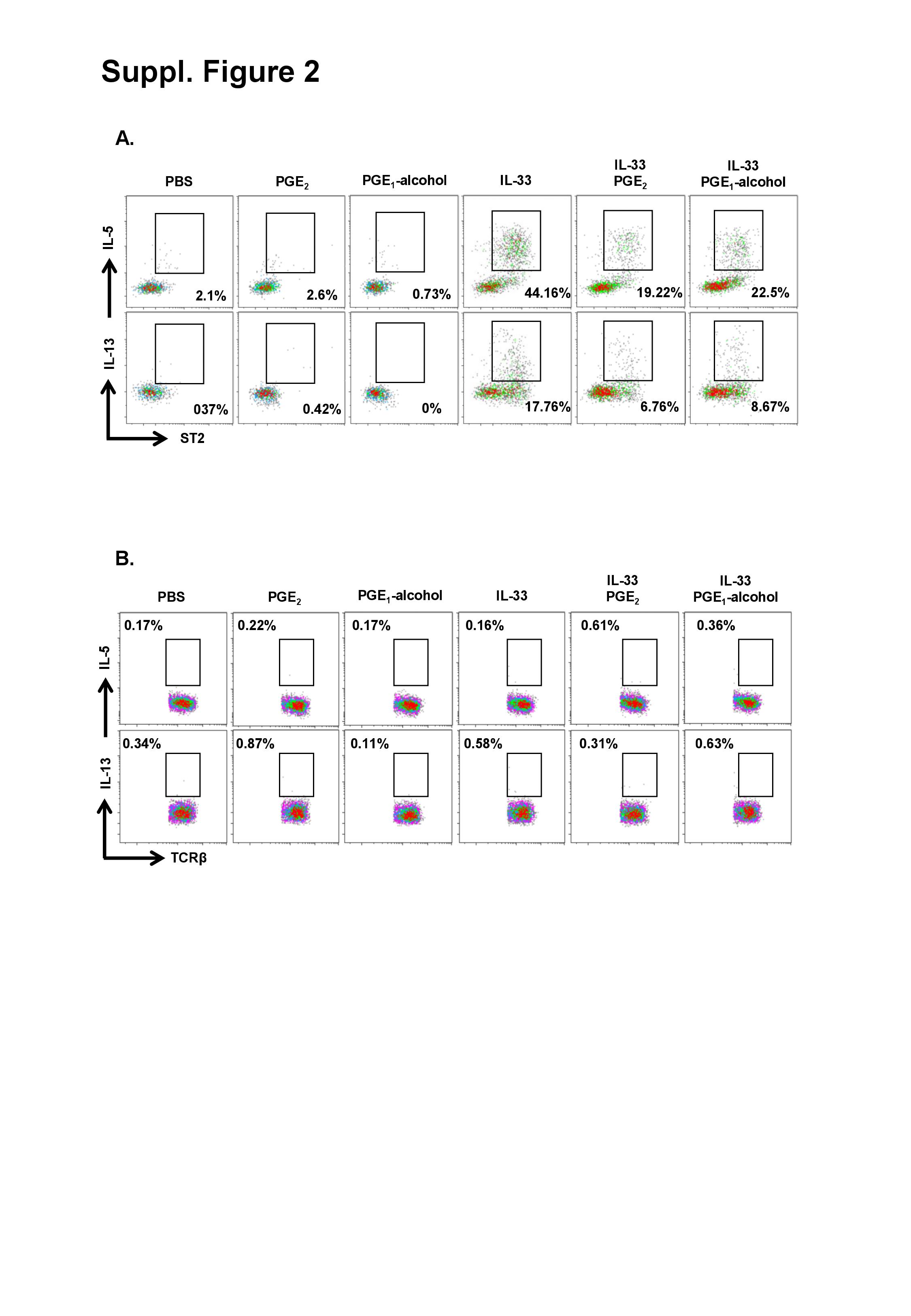

Supplement: Figure S2 — IL-5 and IL-13 production by ILC2 versus CD4+ T cells in the IL-33-induced asthma models. Airway inflammation was induced as described in Figure 3. IL-5 and IL-13 production was analyzed by intracellular staining after gating on Lin-CD45+ST2+ ILC2 (A) or TCRβ+ (B) cells. Representative dot plots are shown. [file image_2.jpg]

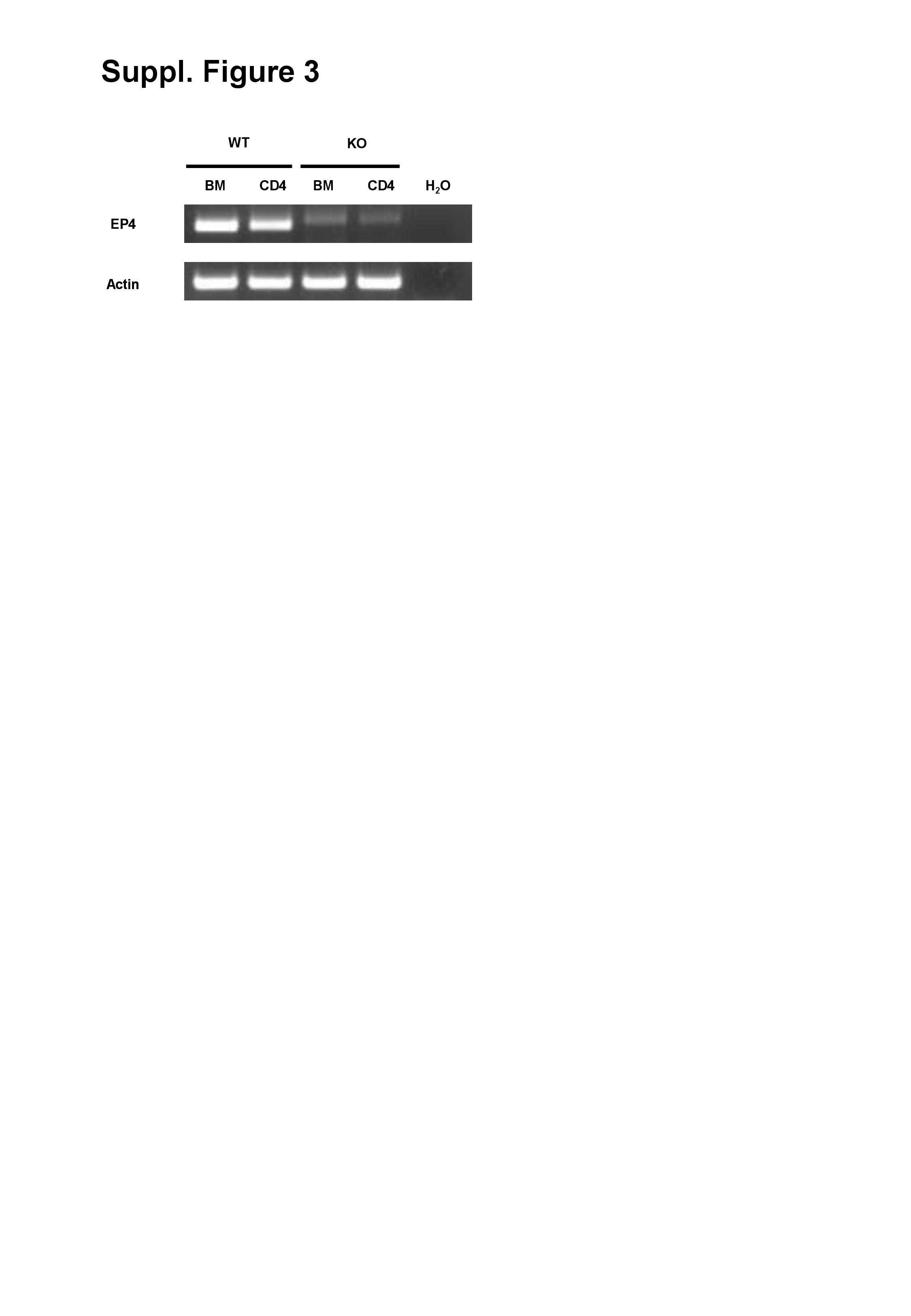

Supplement: Figure S3 — Ptger4 mRNA expression in total bone marrow cells and CD4+ T cells from wild-type (WT) and Ptger4flox/flox-Vav-Cre (KO) mice as determined by RT-PCR. [file image_3.jpg]

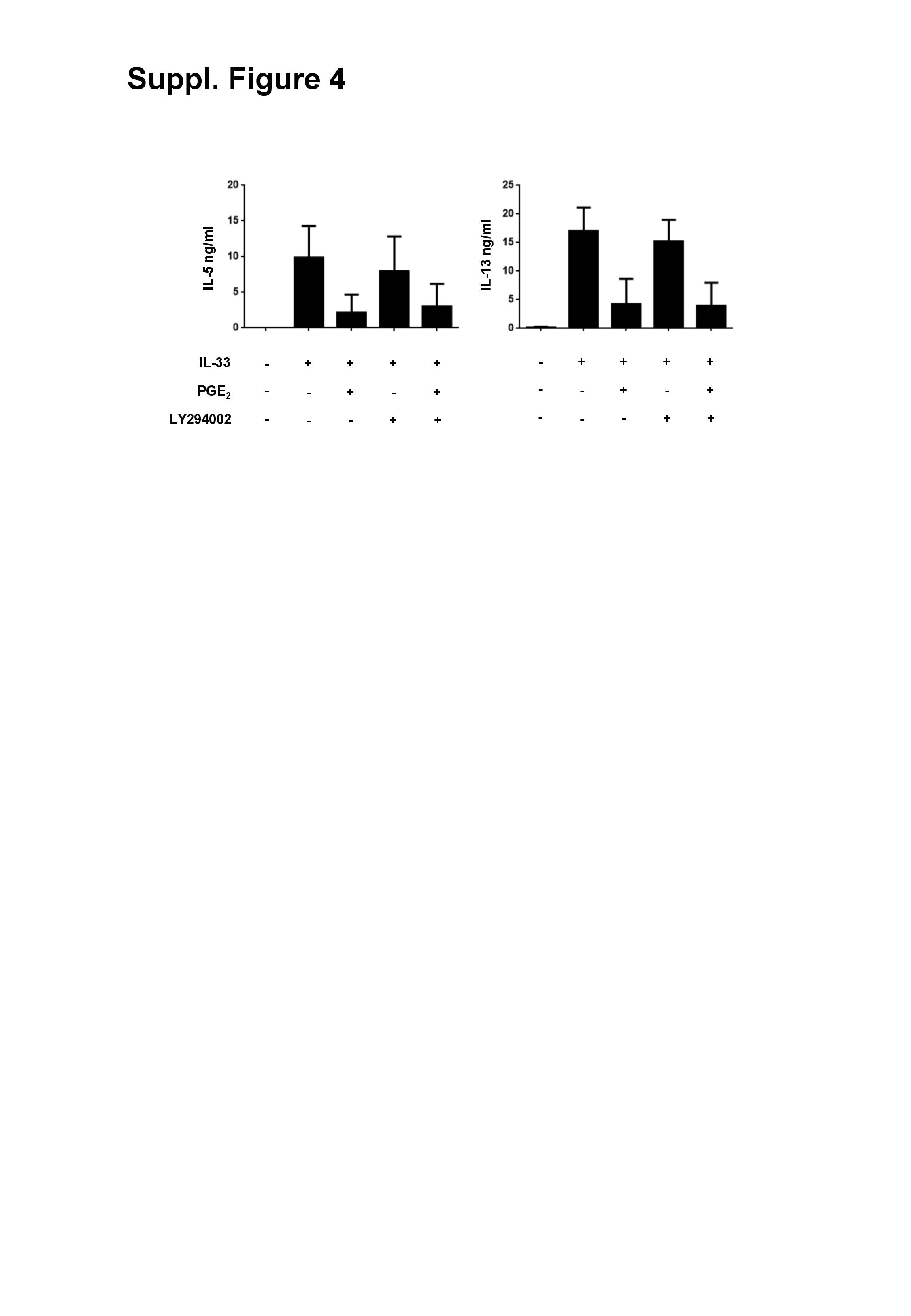

Supplement: Figure S4 — Independence of PGE2-mediated inhibition on PI3K activation. ILC2 cells were stimulated with IL-33 (20 ng/ml) in the presence or absence of PGE2 (10 nM) with or without the addition of PI3K inhibitor LY294002 (10 µM). IL-5 and IL-13 levels in the supernatant were measured by ELISA. [file image_4.jpg]
